# Predictive Effect of Renal Function on Clinical Outcomes in Older Adults With Acute Myocardial Infarction: Results From an Observational Cohort Study in China

**DOI:** 10.3389/fcvm.2021.772774

**Published:** 2021-12-06

**Authors:** Hui Gao, Hui Peng, Aidong Shen, Hui Chen, Hongwei Li

**Affiliations:** ^1^Department of Cardiology, Cardiovascular Center, Beijing Friendship Hospital, Capital Medical University, Beijing, China; ^2^Department of Internal Medical, Medical Health Center, Beijing Friendship Hospital, Capital Medical University, Beijing, China; ^3^Beijing Key Laboratory of Metabolic Disorder Related Cardiovascular Disease, Beijing, China

**Keywords:** estimated glomerular filtration rate, chronic kidney disease, acute coronary syndrome, nomograms, long-term outcomes

## Abstract

**Background:** The impact of estimated glomerular filtration rate (eGFR) on the risk of death and cardiovascular events in individuals with acute myocardial infarction (AMI) is less well established, particularly in the old Chinese population. The aim of this study was to investigate the association of eGFR with clinical outcomes among older subjects with AMI. We further developed a nomogram for the prediction of 1- and 3-year survival in this population.

**Methods:** A cohort of 2,366 AMI subjects aged over 60 years in 2013–2020 were enrolled in the Cardiovascular Center of Beijing Friendship Hospital Database (CBD) Bank. Outcomes including cardiovascular (CV) death, all-cause death, non-fatal myocardial infarction (MI), non-fatal stroke, revascularization, and cardiac rehospitalization were collected overall and by eGFR category at baseline. eGFR was estimated by the Chronic Kidney Disease Epidemiology Collaboration equation (CKD-EPI). Subjects were categorized into four groups according to quartiles of eGFR: ≤ 63.02, 63.03–78.45, 78.46–91.50, >91.51 ml/min/1.73 m^2^. Hazard ratios (HRs), corresponding 95% confidence intervals (CIs) as well as the nomogram were assessed using Cox regression models. Validation of the nomogram was estimated by discrimination and calibration.

**Results:** Incidence rates and multivariable-adjusted hazard ratios of CV and all-cause death decreased significantly across quartiles of eGFR over a median follow-up time of 36.7 months. In adjusted analysis, compared with eGFR ≤ 63.02 ml/min/1.73 m^2^, patients with eGFR of 63.03–78.45, 78.46–91.50, >91.51 ml/min/1.73 m^2^ experienced decreased risks of CV death [respective HRs of 0.58 (95% CI, 0.38–0.90), 0.61 (95% CI, 0.38–0.99), and 0.48 (95% CI, 0.25–0.90); all *p* < 0.05] and all-cause death [respective HRs of 0.64 (95% CI, 0.47–0.88), 0.61 (95% CI, 0.42–0.88), and 0.54 (95% CI, 0.35–0.84); all *p* < 0.05]. Age, eGFR quartiles, BMI, glycated hemoglobin, LVEF, LM/multi-vessel disease, angiotensin-converting enzyme inhibitors (ACEIs), or angiotensin receptor blockers (ARBs) prescribed at discharge were associated with all-cause death. The developed model predicted 1- and 3-year probability of survival, which performed well in both discrimination and calibration.

**Conclusion:** In older patients with AMI, early identification of eGFR reduced and cardiovascular risks management may prevent poor clinical outcomes.

## Introduction

Chronic kidney disease (CKD) remains a major cause of public health problems in the general population (1, 2). Impairment of kidney function is a risk factor for cardiovascular (CV) events (3). Several studies have suggested that patients who have known coronary artery disease with only mild renal insufficiency tend to have higher long-term mortality (4–7). One of the underlying mechanisms is the accumulation of metabolic products that result in increased arterial stiffness (8). Patients with CKD have accelerated atherosclerosis that leads to increased risk of CV outcomes and mortality, especially in older adults (9–13). Although this population represents a majority of adults living with CKD, there are limited data about the influence of renal function on clinical adverse events in those suffered from acute myocardial infarction (AMI).

The glomerular filtration rate (GFR) is regarded as the most appropriate variable for quantifying renal function (14). Most investigations have concentrated on the association between GFR and outcomes for adults in the general (15). Researches demonstrate the prediction of GFR for increased mortality in individuals after AMI (16, 17). Other studies indicated that reduced eGFR had no strong association with total mortality (18). Besides, a British study showed that the association of mild-to-moderate decreased GFR with CV mortality was explicated by other risk factors, including hyperglycemia, hypertension, and hyperlipemia (19). The effect of eGFR on mortality in older patients who experience AMI has not been well established.

In this analysis, we probed to the probable association between differential clinical outcomes and renal function in older subjects with AMI. Furthermore, we examine the predictive value of eGFR for all-cause mortality in this group of people.

## Patients and Methods

### Study Design and Participants Enrolment

The data were obtained from the Cardiovascular Center of Beijing Friendship Hospital Database (CBD) Bank. A total of 5,170 AMI (in accordance with the Fourth Universal Definition of Myocardial Infarction) patients between January 2013 and August 2020 were involved in the CBD Bank. Patients were not selected if any of them (1) aged younger than 60 years; (2) missing baseline creatinine levels; (3) without coronary angiography; or (4) undocumented medical record or long-term results. Finally, 2,366 participants aged over 60 years were recruited in our analysis. This retrospective study was permitted by the institutional review board of Beijing Friendship Hospital affiliated to Capital Medical University. All methods were carried out in conformity with the ethical standards of the institutional and the Declaration of Helsinki.

### Renal Function Assessment

The level of serum creatinine (Scr) was measured at hospital admission before percutaneous coronary intervention (PCI). The renal function was assessed based on the eGFR. eGFR was calculated by using the CKD-EPI: one unit of eGFR is equal to a × (Scr/b)^c^ × (0.993)^age^, where a is 144 for women and 141 for men, b is 0.7 for women and 0.9 for men, and c is −0.329 if female and Scr lessen than or equal to 0.7 mg/dl (62 umol/L); c is −1.209 if female and Scr >0.7 mg/dl (62 umol/L); c is −0.411 if male and Scr lessen than or equal to 0.9 mg/dl (80 umol/L), and c is −1.209 if male and Scr >0.9 mg/dl (80 umol/L) (20).

### Data Collections and Definitions

Demographic, clinical (e.g., hypertension, diabetes, stroke, and dyslipidemia), biochemical (e.g., cholesterol, glycated hemoglobin, and cardiac enzymes), and echocardiographic [e.g., left ventricular ejection fraction (LVEF) values was measured through Simpson's way] data were obtained in all participants. Coronary angiography was performed in this population by interventional teams according to standard clinical practice. Use of medications at discharge was recorded: aspirin, P2Y12 inhibitors, beta-blockers, ACEIs or ARBs, and statins. Cardiovascular (CV) death, all-cause death, non-fatal myocardial infarction (MI), non-fatal stroke, revascularization, and hospitalization due to heart failure were collected for a median duration of 36.7 months. All individuals were followed up via the telephone by trained clinicians. Outcomes were obtained from both electronic medical records and telephone follow-up.

The definition of AMI is in accordance with the Fourth Universal Definition of Myocardial Infarction during the study period (21). Only patients who adhered to type 1 AMI were enrolled in this study. Diabetes was defined as a previous diagnosis, use of antidiabetic drugs, plasma glucose ≥126 mg/dL (7.0 mmol/L) on an empty stomach, or ≥200 mg/dL (11.1 mmol/L) in irregular time. Hypertension was explicated as a previous diagnosis, use of antihypertensive medications, blood pressure ≥140 mmHg in systole or ≥90 mmHg in diastole. Hyperlipidemia was defined as previously diagnosed or a history use of lipid-lowing drugs, or total cholesterol (TC) >200 mg/dL (5.2 mmol/L), low-density lipoprotein cholesterol (LDL-C) >130 mg/dL (3.3 mmol/L), triglycerides (TGs) >150 mg/dL (1.7 mmol/L), high-density lipoprotein cholesterol (HDL-C) <40 mg/dL (1.0 mmol/L) for male or <50 mg/dL (1.3 mmol/L) for female. Non-fatal stroke was explicated as brain disorders lasting >24 h and supported by brain imaging. CV death was defined as death due to cardiovascular causes. All-cause death was explicated as death for any reason. Cardiac rehospitalization was defined as rehospitalization due to heart failure. Revascularization was explicated as any coronary arteries revascularized due to stenosis or occlusion unplanned.

### Statistical Analyses

Statistical analysis of baseline variables was based on a comparison among subgroups according to cut-off points for quartiles of eGFR (≤ 63.02, 63.03–78.45, 78.46–91.50, and >91.51 ml/min/1.73 m^2^). Continuous variables were presented as median (interquartile range) or mean ± SD, and compared using one-way analysis. Categorical variables were presented as counts and percentages and compared using chi-square or Fisher's exact test. The probabilities of events were estimated by Kaplan-Meier (22). Independent prognostic factors were evaluated by Cox proportional hazards analysis. CV mortality, revascularization, cardiac rehospitalization, non-fatal MI, and non-fatal stroke were analyzed through Fine and Gray competition models, due to the confounder from all-cause deaths. Adjusted variables including sex, age, BMI, smoke, patterns of acute myocardial infarction, drink, stroke, systolic blood pressure (SBP), glycated hemoglobin, TG, the peak of CK-MB, and peripheral vascular disease. Univariate and multivariate analyses were applied to estimate the prognostic significance of all-cause death. Analyses were stratified according to age group (60–70 and 70+), patterns of AMI (STEMI and NSTEMI), sex, smoker, diabetes, and hypertension. The comparison group was eGFR ≤ 63.02 ml/min/1.73 m^2^.

A nomogram of all-cause death was performed using Cox proportional hazards regression models. Age, eGFR quartiles, BMI, glycosylated hemoglobin, LVEF, LM/Multi-vessel disease, and ACEI/ARB were finally collected for identification of prognostic factors and design of the predictive model. The final predictive score was established by adding the points for each predictor together. Higher scores illustrate more probability of death among patients. Validation of the nomogram was evaluated by discrimination (C-index) and calibration (calibration curves). The correlation between the incidences of observed results and the probabilities of prediction was illustrated by calibration curves. Decision curve analysis (DCA) was established to represent clinical practicality and net benefit for 1- and 3-year event of all-cause death. All analyses were conducted using R Programming Language and SPSS version 25.0 (IBM Inc, Armonk, and New York). All *p* < 0.05 was considered statistically significant.

## Results

### Baseline Characteristics

Among the 2,366 patients with AMI in the pooled dataset, the mean age (±SD) of 70.6 ± 7.8 years, and eGFR of 76.0 ± 21.7 ml/min/1.73 m^2^. The clinical characteristics of individuals were compared across eGFR quartile levels: eGFR ≤ 63.02 (Quartile 1, *n* = 592), 63.03–78.45 (Quartile 2, *n* = 592), 78.46–91.50 (Quartile 3, *n* = 591), >91.51 (Quartile 4, *n* = 591) ml/min/1.73 m^2^. Compared with other three groups, subjects in Quartile 1 were less likely to be male, smoker, drinker, and discharged on aspirin. Patients in Quartile 1 had a lower tendency to present as STEMI than NSTEMI, while they had higher systolic blood pressure at baseline and length of stay. In this cohort, we identified individuals with lower eGFR were more likely to be old and have LM or multi-vessel disease. The prevalence of co-morbidities such as hypertension, peripheral arterial disease, diabetes, and stroke increased gradually with decreasing eGFR. The creatine kinase-MB isoenzyme (CK-MB) peak value, HDL-C, and LVEF were lower in Quartile 1 (all *p* < 0.05). Median values of CK-MB were 52.5, 69.9, 88.4, and 77.0 ng/ml in Quartiles 1, 2, 3, and 4, respectively (*p* = 0.002). Subjects in Quartile 1 had a decreased LDL-C values, although these differences were not statistically significant. The level of hemoglobin and median LVEF was lower in Quartile 1 when compared with the other three groups (*p* < 0.001) (Table 1).

**Table 1:** Baseline characteristics of the study population.

	**eGFR quartiles (ml/min per 1.73 m** ^**2**^ **)**				***P* value**
	**≤63.02 (*N* = 592)**	**63.03–78.45 (*N* = 592)**	**78.46–91.50 (*N* = 591)**	**>91.51 (*N* = 591)**	
**Demographics**					
Male	325 (54.9)	385 (65.0)	402 (68.0)	491 (83.1)	<0.001
Age	75.2 ± 7.3	71.5 ± 7.8	69.9 ± 7.5	66.0 ± 5.3	<0.001
STEMI	250 (42.2)	275 (46.5)	304 (51.4)	281 (47.5)	0.023
BMI, kg/m^2^	25.0 ± 3.6	25.2 ± 3.2	25.1 ± 3.4	24.9 ± 3.4	0.408
SBP, mmHg	135.7 ± 23.7	130.7 ± 21.1	128.3 ± 20.5	126.4 ± 20.6	<0.001
DBP, mmHg	73.2 ± 12.3	73.1 ± 11.8	72.7 ± 12.2	72.4 ± 12.3	0.197
**Past medical history**					
Current smokers	148 (25.0)	181 (30.6)	207 (35.0)	289 (48.9)	0.002
Alcohol use	127 (21.5)	176 (29.7)	185 (31.3)	249 (42.1)	<0.001
Hypertension	488 (82.4)	425 (71.8)	387 (65.5)	348 (58.9)	<0.001
Diabetes	240 (40.5)	205 (34.6)	202 (34.2)	204 (34.5)	0.036
Dyslipidemia	283 (47.8)	272 (45.9)	259 (43.8)	273 (46.2)	0.448
Peripheral vascular disease	55 (9.3)	45 (7.6)	30 (5.1)	31 (5.2)	0.002
Stroke	155 (26.2)	134 (22.6)	95 (16.1)	76 (12.9)	<0.001
Hemodialysis	47 (7.9)	2 (0.3)	1 (0.2)	1 (0.2)	<0.001
**Laboratory values**					
TC, mmol/L	4.2 ± 1.0	4.3 ± 0.9	4.3 ± 0.9	4.3 ± 1.0	0.251
TG, mmol/L	1.5 ± 0.9	1.6 ± 0.8	1.5 ± 0.8	1.4 ± 0.8	0.025
HDL-C, mmol/L	1.0 ± 0.2	1.1 ± 0.2	1.1 ± 0.2	1.1 ± 0.2	0.026
LDL-C, mmol/L	2.4 ± 0.7	2.5 ± 0.6	2.5 ± 0.7	2.4 ± 0.7	0.157
Hemoglobin, g/L	123.6 ± 18.8	132.7 ± 17.3	133.4 ± 16.8	138.4 ± 15.2	<0.001
Anemia	168 (28.3)	68 (11.4)	76 (12.8)	47 (7.9)	<0.001
Glycated hemoglobin, %	6.6 ± 1.4	6.5 ± 1.4	6.5 ± 1.4	6.5 ± 1.4	0.087
Peak of CK-MB, ng/ml	52.5 (6.8, 109.0)	69.9 (10.9, 110.7)	88.4 (12.0, 141.0)	77.0 (11.4, 121.0)	0.002
Peak of TnI, ng/ml	6.8 (0.9, 12.0)	6.2 (1.1, 12.0)	10.0 (1.5, 14.4)	7.2 (0.9, 12.0)	0.405
**Echocardiographic values**					
LVEF, %	57.2 ± 10.7	59.6 ± 9.6	58.5 ± 9.3	59.7 ± 8.9	<0.001
**Angiography values**					
LM/ Multi-vessel	498 (84.1)	469 (79.2)	444 (75.1)	440 (74.5)	<0.001
Pre-PCI TIMI 0/1 flow	188 (31.8)	180 (30.4)	230 (38.9)	210 (35.5)	0.023
**Medications at discharge**					
Aspirin	548 (92.6)	564 (95.3)	566 (95.8)	564 (95.4)	0.027
P2Y12 inhibitors	521 (88.0)	519 (87.7)	538 (91.0)	520 (88.0)	0.571
ACEI or ARB	398 (67.2)	419 (70.8)	405 (68.5)	374 (63.3)	0.102
Beta-blockers	435 (73.5)	430 (72.6)	442 (74.8)	432 (73.1)	0.902
Statins	529 (89.4)	536 (90.5)	535 (90.5)	532 (90.0)	0.721
Length of stay, days	9.0 (7.0, 12.0)	8.0 (6.0, 10.0)	8.0 (6.0, 10.0)	8.0 (6.0, 10.0)	0.008

*Values are presented as mean ± SD, median (IQR) or number (%)*.

*STEMI, ST-elevation myocardial infarction; BMI, body mass index; SBP, systolic blood pressure; DBP, diastolic blood pressure; TG, triglyceride; HDL-C, high-density lipoprotein cholesterol; PCI, percutaneous coronary intervention; ACEI or ARB, angiotensin-converting enzyme inhibitor/angiotensin receptor blocker; CCB, calcium channel blocker; eGFR, estimated glomerular filtration rate; TC, total cholesterol; LDL-C, low-density lipoprotein cholesterol; MI, myocardial infarction; LVEF, left ventricular ejection fraction; LM, left main coronary artery*.

### Outcomes

**Figure 1:**
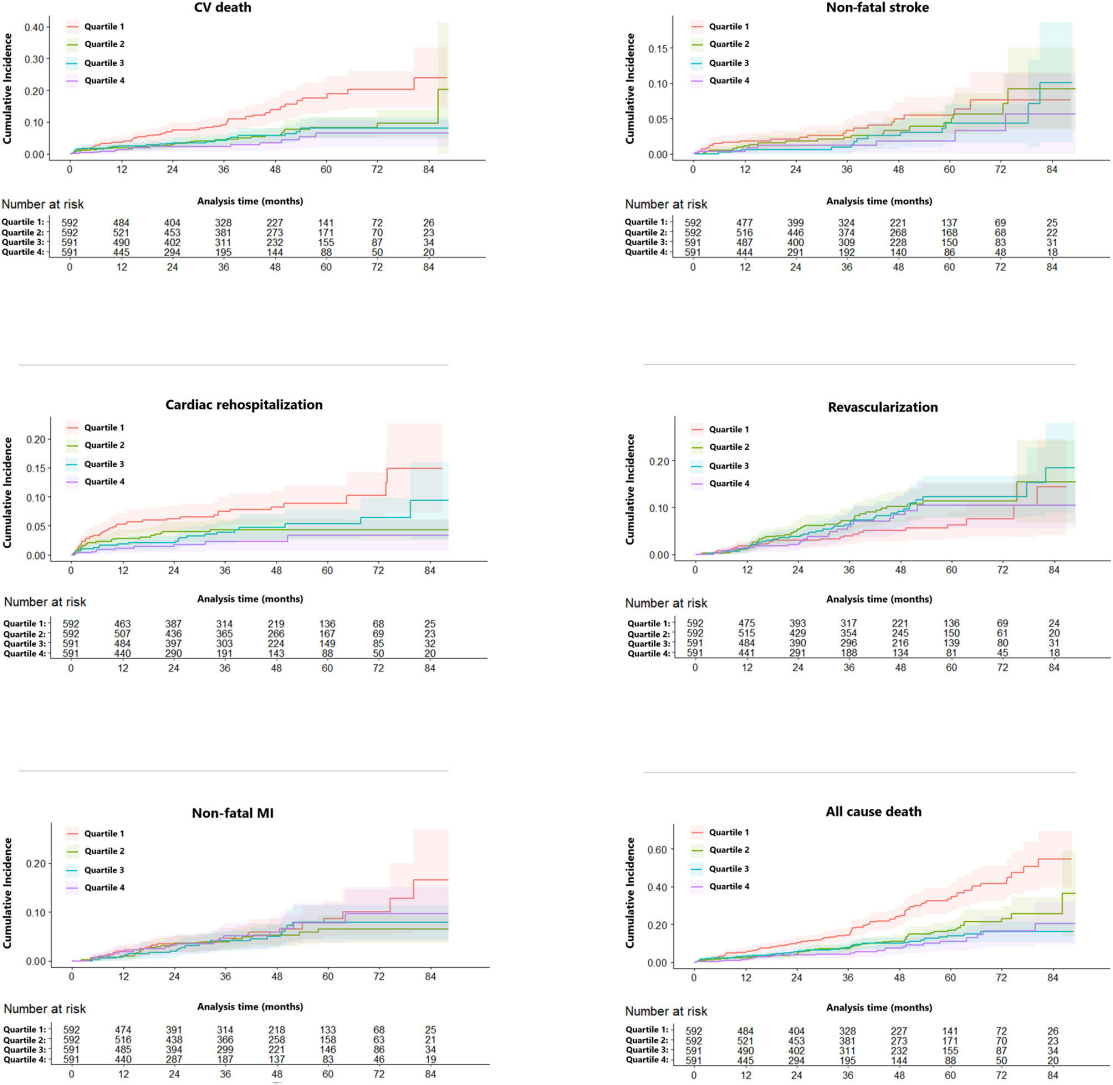
Competing risk regression curves for CV death, non-fatal MI, non-fatal stroke, cardiac rehospitalization, and revascularization of the eGFR ≤ 63.02 (Quartile 1, red line), 63.03–78.45 (Quartile 2, green line), 78.46–91.50 (Quartile 3, blue line), >91.51 (Quartile 4, purple line) ml/min/1.73 m^2^. CV, cardiovascular; MI, myocardial infarction; eGFR, estimated glomerular filtration rate.

Over a median follow-up time of 36.7 months, 260 (11.0%) patients died. Subjects with lower eGFR were more probably to suffer from cardiovascular outcomes except for revascularization (Table 2). Figure 1 highlights the unadjusted Kaplan-Meier estimates of cardiac rehospitalization, CV and all-cause mortality decreased with increasing eGFR group (all *p* < 0.001). Univariable and multivariable competing risk models were constructed to compare risk between groups for clinical outcomes. Using the 1st quartile of eGFR as the referent group, the HR decreased with rising eGFR quartile groups for CV death on both unadjusted and adjusted competing risk modeling (*p* < 0.05). In the unadjusted model, increasing of eGFR was associated with decreasing HR for cardiac rehospitalization, while adjusting for age, sex, and other confounders, HR decreased statistically significant only in Quartile 4 (adjusted HR: 0.34; 95% CI: 0.15 to 0.77; *p* = 0.009). The association between eGFR quartile groups and non-fatal stroke, non-fatal MI, revascularization was not observed in our study (Table 2).

**Table 2:** Competing risk model of clinical outcomes.

	**Events (%)**	**Unadjusted HR (95% CI)**	***p* value**	**Adjusted HR (95% CI)**	***p* value**
**CV death**					
≤ 63.02	64 (10.8)	Ref	-/-	Ref	-/-
63.03–78.45	32 (5.4)	0.46 (0.30, 0.71)	<0.001	0.58 (0.38, 0.90)	0.017
78.46–91.50	28 (4.7)	0.44 (0.28, 0.70)	<0.001	0.61 (0.38, 0.99)	0.048
>91.51	14 (2.4)	0.29 (0.16, 0.51)	<0.001	0.48 (0.25, 0.90)	0.024
**Cardiac rehospitalization**					
≤ 63.02	42 (7.1)	Ref	-/-	Ref	-/-
63.03–78.45	22 (3.7)	0.50 (0.30, 0.85)	0.010	0.57 (0.32, 1.02)	0.060
78.46–91.50	22 (3.7)	0.53 (0.31, 0.89)	0.016	0.60 (0.33, 1.09)	0.096
>91.51	10 (1.7)	0.28 (0.14, 0.56)	<0.001	0.34 (0.15, 0.77)	0.009
**Non-fatal MI**					
≤ 63.02	30 (5.1)	Ref	-/-	Ref	-/-
63.03–78.45	24 (4.1)	0.77 (0.45, 1.32)	0.340	0.78 (0.45, 1.35)	0.370
78.46–91.50	23 (3.9)	0.81 (0.47, 1.39)	0.450	0.85 (0.47, 1.53)	0.600
>91.51	21 (3.6)	1.00 (0.57, 1.76)	0.980	1.08 (0.57, 2.03)	0.800
**Revascularization**					
≤ 63.02	25 (4.2)	Ref	-/-	Ref	-/-
63.03–78.45	43 (7.3)	1.71 (1.04, 2.79)	0.033	1.54 (0.93, 2.55)	0.091
78.46–91.50	39 (6.6)	1.69 (1.02, 2.79)	0.041	1.44 (0.84, 2.47)	0.180
>91.51	23 (3.9)	1.36 (0.77, 2.41)	0.290	1.04 (0.57, 1.92)	0.880
**Non-fatal stroke**					
≤ 63.02	22 (3.7)	Ref	-/-	Ref	-/-
63.03–78.45	20 (3.4)	0.89 (0.48, 1.63)	0.710	0.92 (0.50, 1.72)	0.820
78.46–91.50	13 (2.2)	0.62 (0.31, 1.25)	0.190	0.69 (0.33, 1.46)	0.330
>91.51	8 (1.4)	0.52 (0.23, 1.18)	0.120	0.59 (0.24, 1.43)	0.240
**All cause death**					
≤ 63.02	118 (19.9)	Ref	-/-	Ref	-/-
63.03–78.45	65 (11.0)	0.49 (0.36, 0.67)	<0.001	0.64 (0.47, 0.88)	0.006
78.46–91.50	49 (8.3)	0.41 (0.29, 0.57)	<0.001	0.61 (0.42, 0.88)	0.009
>91.51	28 (4.7)	0.32 (0.21, 0.48)	<0.001	0.54 (0.35, 0.84)	0.007

*Adjusted factors included sex, age, BMI, smoke, patterns of acute myocardial infarction, drink, stroke, SBP, glycated hemoglobin, peak of CK-MB, peripheral vascular disease, and TG*.

**Figure 2:**
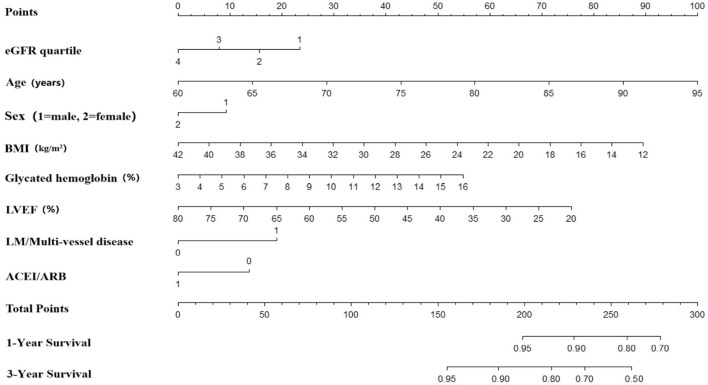
Instructions for Using the Nomogram. Draw a line perpendicular from the corresponding axis of each risk factor until it reaches the top line labeled “Points.” Sum up the number of points for all risk factors then draw a line descending from the axis labeled “Total Points” until it intercepts each of the survival axes to determine 1- and 3-year survival probabilities. For eGFR quartile, 1 = Quartile 1 (eGFR ≤ 63.02 ml/min/1.73 m^2^), 2 = Quartile 2 (eGFR 63.03–78.45 ml/min/1.73 m^2^), 3 = Quartile 3 (eGFR 78.46–91.50 ml/min/1.73 m^2^), 4 = Quartile 4 (eGFR > 91.51 ml/min/1.73 m^2^). For sex, 1 = male and 2 = female. For LM/Multi-vessel disease, 0 = no and 1 = yes. For ACEI/ARB, 0 = no use and 1 = use. eGFR, estimated glomerular filtration rate; BMI, Body Mass Index; LVEF, left ventricular ejection fraction; LM, LM, left main coronary artery; ACEI or ARB, angiotensin-converting enzyme inhibitor/angiotensin receptor blocker.

**Figure 3:**
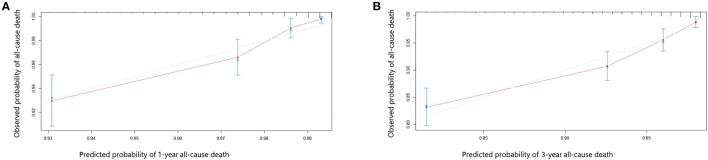
Calibration curves showing the probability of 1- year **(A)** and 3-year **(B)** all-cause death between the nomogram prediction and the actual observation.

**Figure 4:**
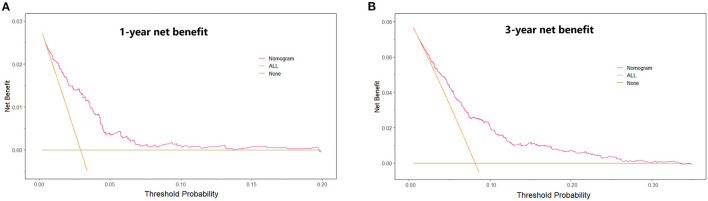
Decision curve analysis. The net benefit of using a “Nomogram” model to predict the 1- year **(A)** and 3-year **(B)** event of all-cause death as compared with strategies of “ALL” (assume all patients will have death for any reason), or “None” (assume death for any reason occurred in no patients) for different thresholds.

Univariate and multivariate Cox proportional hazards regression analyses for all-cause death were performed in Table 3. In the univariate Cox proportional hazards regression model, several predictive factors correlated with all-cause death were eGFR quartiles, age, BMI, diabetes, stroke, TC, TG, LDL-C, glycated hemoglobin, LVEF, LM or multi-vessel disease, and drugs at discharge (aspirin, P2Y12 inhibitors, ACEI or ARB, and statins). Age, eGFR quartiles, BMI, TG, LVEF, aspirin, and ACEI or ARB prescribed at discharge had a high correlation (all *p* < 0.001). Adjusting for confounding factors, age, eGFR quartiles, BMI, glycated hemoglobin, LVEF, LM or multi-vessel disease, ACEI or ARB were regarded as independent predictors for the incidence of all-cause death in AMI individuals and were included in the final model to build the nomogram (Figure 2). The nomogram can be used to predict the probability of a patient's death at 1 year or 3 years. For a given patient, locate the patient's variables and rule a line straight up to the top line of the nomogram, labeled “Points,” to establish the score associated with each of the eight variables. Then add the scores for all variables together and locate on the “Total Points.” A vertical line dropping to the bottom of the survival-probability axis revealed the probability of 1-year and 3-year survival for each person. The C-index was 0.739 (95% CI 0.71–0.77) in this model, reflecting the availability of discrimination. The calibration curves were also displayed based on this model in Figure 3. The probabilities of 1- and 3-year survival made by the model were close to the actual outcomes, which showed good consistency between the predicted outcomes and the observed outcomes. Besides, DCA compared the net benefits of 1- and 3-year of all-cause death, which revealed the good potential clinical effect of the predictive model (Figure 4).

**Table 3:** Independent predictors of all-cause death.

	**Univariate**	***p* value**	**Multivariate**	***p* value**
	**HR (95% CI)**		**Adjusted HR (95% CI)**	
**eGFR quartiles**
≤ 63.02	Reference		Reference	
63.03–78.45	0.49 (0.36, 0.67)	<0.001	0.68 (0.50, 0.93)	0.015
78.46–91.50	0.40 (0.29, 0.57)	<0.001	0.63 (0.45, 0.89)	0.009
>91.51	0.32 (0.21, 0.48)	<0.001	0.62 (0.39, 0.98)	0.039
**Demographics**
Female	1.02 (0.79, 1.33)	0.834	0.89 (0.67, 1.18)	0.429
Age	1.08 (1.07, 1.10)	<0.001	1.06 (1.05, 1.08)	<0.001
STEMI	1.09 (0.85, 1.40)	0.459		
BMI, kg/m^2^	0.90 (0.87, 0.94)	<0.001	0.93 (0.90, 0.97)	0.001
SBP, mmHg	1.00 (0.99, 1.01)	0.079		
DBP, mmHg	0.99 (0.98, 1.00)	0.682		
**Past medical history**
Current smokers	1.04 (0.80, 1.34)	0.766		
Alcohol use	0.76 (0.57, 1.01)	0.062		
Hypertension	1.31 (0.99, 1.74)	0.051		
Diabetes	1.35 (1.06, 1.73)	0.015		
Dyslipidemia	0.86 (0.67, 1.11)	0.258		
Peripheral vascular disease	1.52 (0.97, 2.38)	0.065		
Stroke	1.45 (1.09, 1.92)	0.010	1.15 (0.86, 1.53)	0.336
**Laboratory values**
TC, mmol/L	0.80 (0.70, 0.92)	0.002	0.70 (0.46, 1.05)	0.088
TG, mmol/L	0.67 (0.56, 0.82)	<0.001	0.87 (0.70, 1.07)	0.188
HDL-C, mmol/L	0.61 (0.36, 1.02)	0.063		
LDL-C, mmol/L	0.81 (0.68, 0.98)	0.029	1.45 (0.85, 2.48)	0.167
Glycated hemoglobin, %	1.10 (1.02, 1.19)	0.009	1.11 (1.03, 1.19)	0.006
Peak of CK-MB, ng/ml	0.99 (0.99, 1.00)	0.085		
Peak of TnI, ng/ml	0.99 (0.98, 1.00)	0.121		
**Echocardiographic values**
LVEF, %	0.02 (0.00, 0.06)	<0.001	0.06 (0.02, 0.18)	<0.001
**Angiography values**
LM/Multi-vessel	1.97 (1.33, 2.91)	0.001	1.56 (1.04, 2.34)	0.028
**Medications at discharge**
Aspirin	0.40 (0.26, 0.61)	<0.001	0.71 (0.42, 1.20)	0.205
P2Y12 inhibitors	0.70 (0.49, 0.98)	0.041	0.92 (0.62, 1.38)	0.708
ACEI or ARB	0.63 (0.49, 0.82)	<0.001	0.76 (0.58, 0.99)	0.047
Beta-blockers	0.78 (0.60, 1.01)	0.067		
Statins	0.61 (0.43, 0.87)	0.006	0.78 (0.51, 1.19)	0.250

*STEMI, ST-elevation myocardial infarction; BMI, body mass index; SBP, systolic blood pressure; DBP, diastolic blood pressure; TG, triglyceride; HDL-C, high-density lipoprotein cholesterol; PCI, percutaneous coronary intervention; ACEI or ARB, angiotensin-converting enzyme inhibitor/angiotensin receptor blocker; CCB, calcium channel blocker; eGFR, estimated glomerular filtration rate; TC, total cholesterol; LDL-C, low-density lipoprotein cholesterol; MI, myocardial infarction; LVEF, left ventricular ejection fraction; LM, left main coronary artery*.

**Figure 5:**
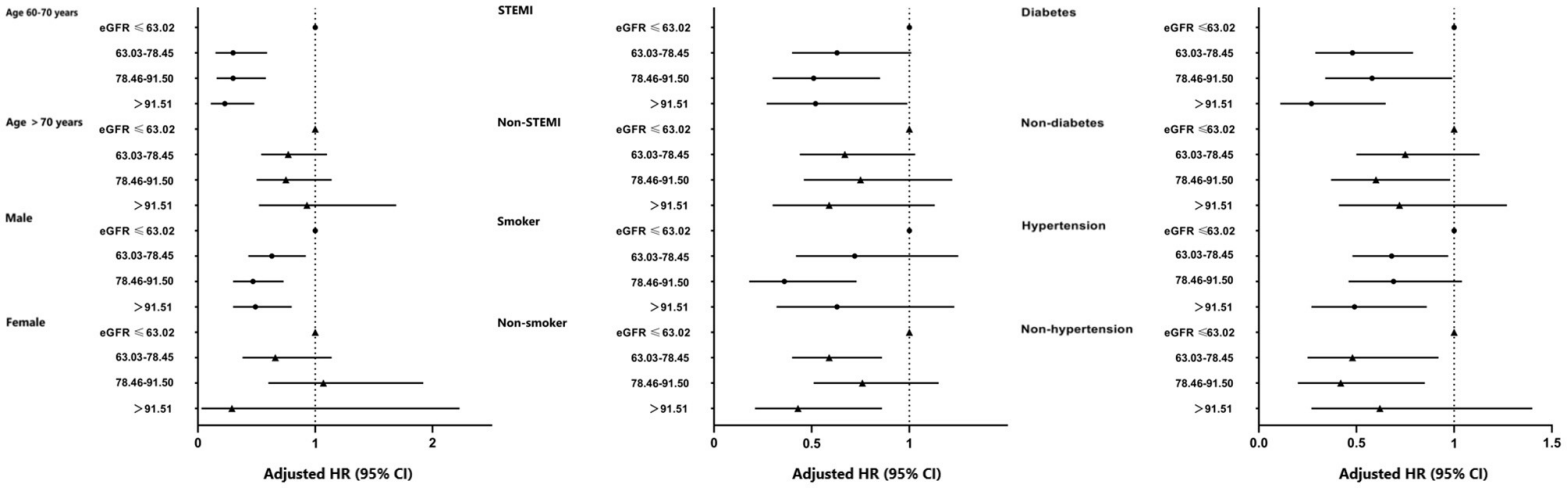
Forest-plot of all-cause death according to different subgroups. Adjusted model included sex, age, BMI, smoke, patterns of acute myocardial infarction, drink, stroke, SBP, glycated hemoglobin, peak of CK-MB, peripheral vascular disease, and TG. BMI, body mass index; eGFR, estimated glomerular filtration rate; STEMI, ST-elevation myocardial infarction; SBP, systolic blood pressure; TG, triglycerides; HR, hazard ratio; CI, confidence interval; Ref., reference (eGFR ≤ 63.02 ml/min/1.73 m^2^ group).

Subgroup investigations were analyzed based on age, sex, the pattern of AMI, smoker, diabetes, and hypertension (Figure 5). The risk of all-cause death was lower for men (all *p* < 0.05) and age between 60 and 70 years (all *p* < 0.001) in other three groups compared with the 1st quartile of eGFR. Besides, we identified the prediction of quartiles of eGFR on all-cause death was effective in most subgroups, except for women, aged over 70 years, and presented with NSTEMI.

## Discussion

The difference between kidney function and long-term clinical outcomes in subjects aged over 60 years is less well studied. In this study, we found independent associations of increased eGFR with reduced all-cause mortality in the AMI population aged over 60 years, especially male patients aged 60–70 years, and with diabetes. There seemed to be a gradient of effect, with risks greater in Quartile 1 than other three groups. We found evidence of impaired renal function is strongly associated with male gender, older age, reduced LVEF, raised LM or multi-vessel disease, history of alcohol drinking, stroke, and hypertension. We further developed a simple and practicable nomogram model for predicting 1- and 3-year survival, which could be consulted to inform patients about their future risk. In addition, clinical risk factors included in this model could be obtained easily.

Renal dysfunction is an essential associate of cardiovascular risk factors. Patients with 3–4 stages of CKD have a higher risk of cardiac death than subjects with normal renal function (23). EGFR declines may be following by the increase of severity of coronary stenosis. Several observational studies have reported that the prevalence of 1-year composite MACE was increased as renal insufficiency aggravated in the AMI population (24). A prospective study revealed that decreased eGFR is considerably related to a higher 1-year mortality rate in AMI subjects (25). EGFR may correlate to the long-term production of creatinine, which was associated with hemodynamic control in renal blood flow. The variation of eGFR may reflect the atherosclerotic disease of renal arteries and deterioration of renal function (26). Vascular calcification is prevalent in subjects with decreasing eGFR and is one of the predictive factors of cardiovascular death (27). Some inconsistencies were found in the recent literature. One study revealed that there was no significant distinction in the occurrence of stroke or myocardial re-infarction in subjects with a high risk of MI during a median 23 months follow-up (28). A Japanese study revealed no difference in the risk of death or stroke with reduced eGFR (45–60 ml/min/1.73 m^2^) compared to normal renal function (29). In our study, we did not observe decreased risk associated with eGFR above 63.02 ml/min/1.73 m^2^ in comparison to the reference group for cardiac rehospitalization, non-fatal MI, revascularization, and non-fatal stroke which were consistent with previous studies. A slight reduction of eGFR may result from pathologies of geriatrics. One analysis enrolled 169,826 subjects demonstrated that the risk of in-hospital mortality in populations with kidney failure was greater in young than the old after primary PCI. Moukarbel et al., however, did not observe a similar result (28). In general, lower eGFR was associated with adverse outcomes in older patients. Traditional and non-traditional risk factors become increasingly prevalent in those populations, which may weaken the protection of a high level of eGFR.

In the present study, STEMI was more common than NSTEMI in patients with higher eGFR classification. These similar observations were previously shown by others (30, 31). The number of involved vessels gradually increased following from Quartile 1 to Quartile 4 in our angiographic findings. Renal insufficiency may result in modifications in plasma components and endothelial structure which could potentially accelerate vascular damage and inflammatory reaction (32). The level of hs-CRP was decreased with higher eGFR (30). And studies have shown that the reduction of uremic toxins in patients with normal renal function is of importance to slow down the pathogenesis of atherosclerosis (33). Compared with patients with reduced renal function, those with normal eGFR were not inclined to suffer from cardiac remodeling and systolic insufficiency. Co-morbidities, for instance, hypertension and diabetes are common in subjects with decreased kidney function (28). In most countries, diabetic kidney disease is common in patients with end-stage renal disease (34). And patients with diabetic kidney disease are especially vulnerable to acute kidney injury due to interstitial fibrosis (34). In our analysis, individuals with lower eGFR were more probably to have higher SBP. In contrast, Masoli et al. found that lower SBP (<120 mmHg) was associated with a greater risk of death in older subjects present with chronic kidney disease 3 or 4 stages (35). Smoking has been identified as a risk factor for cardiovascular disease. Compared with patients in the other three groups, those in Quartile 1 had a lower proportion of current smokers and drinkers. Similar results can be found in several large cohort studies (28, 36). The association between smoking and high eGFR may due to residual confounding factors. As for the feature of patients in our study, subjects in Quartile 1 were more likely to be male and at an earlier age. Conventional risk factors, for instance, dyslipidemia, and obesity were not observed statistically significant differences among eGFR categories in this study. However, a systematic review by Heine et al. investigated the metabolism and functionality of lipoproteins that were likely related to renal impairment (37). There was no significant difference in the usage of lipid-lowering agents in the current study. The effect of lipid-lowering drugs was suspended in subjects who stay with the end-stage renal situation. Transference from atherosclerotic to non-atherosclerotic may contribute to one of the probable explanations (37). It was an interesting tendency of death from any cause across age and sex strata in our analysis. Males as well as aged 60–70 years with eGFR > 63.02 ml/min/1.73 m^2^ showed lower risks for all-cause mortality compared to the ones in Quartile 1 separately, while females or aged over 70 years were not noted. In addition, the prognostic value in elderly patients remains uncertain. The percentage of the male was increasing from Quartile 1 to Quartile 4. A large cohort including 12,636 patients revealed similar findings (30). Longevity as well as the reduction in renal function with aging could be the explanation for the difference in CKD prevalence between men and women (38, 39). Previous studies showed that men experience more reduction of renal function (40). However, the progression of kidney disease might not be slower among women than in men, even though the protective effects of estrogen hormones (41). The level of hemoglobin was different with increased eGFR quartile levels in our analysis. And the prevalence of anemia decreased gradually with increasing eGFR quartile levels. Anemia is a complication of renal insufficiency because these patients may develop iron deficiency which synthesizes in the renal peritubular cells (42). As the kidney function deteriorates, chronic kidney disease patients have to receive dialysis treatment. Then additional iron infusion was needed due to poor production by the kidneys (43).

Ziembicka et al. developed a simple clinical tool to predict blood pressure or kidney function improvement in patients with atherosclerotic renal artery stenosis after stent implantation (44). In this study, we further developed one nomogram for patients with AMI to predict 1-year and 3-year survival. Framingham risk score is an early scoring method to predict cardiovascular events, while poor outcomes were underestimated, especially in CKD patients. Therefore, assessment of the correlation between renal function and adverse cardiovascular events is essential for those. The clinical factors involved in our model including eGFR quartiles, age, sex, BMI, LVEF, LM or multi-vessel disease, ACEI or ARB prescribed at discharge, and glycated hemoglobin could be easily ascertained. The eight variables were all independently associated with all-cause mortality. As an example, a male age 75 years has a calculated eGFR in Quartile 2; is LM/multi-vessel disease; BMI is 26 kg/m^2^; the concentration of glycated hemoglobin is 7%; LVEF is 45%; and prescribed without ACEI/ARB at discharge, will have a total risk score of 212 points, which corresponds to a 1- and 3-year probability of survival of 93 and 82% (**Table 1, Figure 2**). Then calibration curves reflect the predictions established by the model that came close to the actual outcomes. The C-index of the nomogram in our study was as high as 0.739 for all-cause death. Calibration plots indicated a good fit of nomogram prediction and the actual observation. Besides, we observed the model had a larger net benefit in predicting death.

## Study Limitations

Our analyses remained some inevitable limitations. First, this is a single-center retrospective study, the findings should be cautious due to confounding factors and selection bias. Second, the data on proteinuria which may provide more information for further analysis were absent. Third, biochemical tests were measured only once at admission, which could change during the long follow-up period. In addition, the validation of the nomogram model should be verified through an external way.

## Conclusion

Our findings show that CV and all-cause death decrease significantly with increasing values of eGFR in older patients following MI. Management of related cardiovascular risk factors, for instance, smoking, hypertension, and diabetes were considered to be addressed, especially in patients with lower eGFR. The findings emphasize that the importance of renal function monitoring regularly.

## Data Availability Statement

The original contributions presented in the study are included in the article/supplementary materials, further inquiries can be directed to the corresponding author.

## Ethics Statement

The studies involving human participants were reviewed and approved by the institutional review board of Beijing Friendship Hospital affiliated to Capital Medical University.

## Author Contributions

HL designed the protocol of the study. HG draft the manuscript. HG and other authors participated in the collection, interpretation, and analysis of the data. All authors approved the final version for publication.

## Funding

This research was supported by a grant from Beijing Key Clinical Subject Program.

## Conflict of Interest

The authors declare that the research was conducted in the absence of any commercial or financial relationships that could be construed as a potential conflict of interest.

## Publisher's Note

All claims expressed in this article are solely those of the authors and do not necessarily represent those of their affiliated organizations, or those of the publisher, the editors and the reviewers. Any product that may be evaluated in this article, or claim that may be made by its manufacturer, is not guaranteed or endorsed by the publisher.
